# *SGK3* promoter deletion in late-onset hypophosphatemic rickets, a possible genetic cause of the disease

**DOI:** 10.1186/s13023-026-04335-0

**Published:** 2026-03-30

**Authors:** Ahmet Uçar, Najla Albader, Amal Qattan, Huda A. BinEssa, Ebru Mısırlı Özdemir, Mustafa Özdemir, Minjing Zou, Ali S. Alzahrani, Yufei Shi

**Affiliations:** 1 Department of Pediatric Endocrinology & Diabetes, Şişli Hamidiye Etfal Training and Research Hospital, Istanbul, Turkey; 2 Innovation and Research, King Faisal Specialist Hospital & Research Centre, P.O. Box 3354, Riyadh, 11211 Saudi Arabia; 3 Medicine, King Faisal Specialist Hospital & Research Centre, Riyadh, Saudi Arabia

**Keywords:** *SGK3*, *PTH1R*, Promoter, Rickets, Hypophosphataemia

## Abstract

**Background:**

Hypophosphataemic rickets (HR) is a group of rare hereditary renal phosphate wasting disorders caused by mutations in the *PHEX*, *FGF23*, *DMP1*,* ENPP1*,* CLCN5*, SGK3, *SLC9A3R1*,* SLC34A1* or *SLC34A3*.

**Objective:**

To identify the genetic defects in a patient with late-onset hypophosphatemia.

**Subjects:**

A 15-year old boy with hypophosphatemic rickets, his unaffected parents and 7 siblings.

**Design:**

Patients and their family members were initially analyzed for PHEX and FGF23 mutations by PCR-sequencing analysis. Exome and whole-genome sequencing were subsequently performed to identify causative genetic defects.

**Results:**

The patient was normal until the age of 11 years old. He presented with severe rickets, hypophosphatemia with increased renal phosphate wasting, normal serum calcium, 1,25(OH)2D and parathyroid hormone (PTH), and mild elevation of serum FGF23. No mutation was detected in the genes known to cause HR such as PHEX, FGF23, DMP1, ENPP1, CLCN5, SGK3, FAM20C, SLC9A3R1, SLC34A1 or SLC34A3 by exome sequencing. A heterozygous variant c.448 C>T (p.Arg150Cys) in the PTH1R gene was found in the patient, his unaffected father and 5 siblings. The same heterozygous variant was previously reported in human enchondromatosis and caused enchondroma-like lesions in transgenic mice. A large heterozygous deletion of more than 7 Kb in the SGK3 distal promoter was identified in the patient by whole genome sequencing.

**Conclusions:**

SGK3 distal promoter deletion is likely the candidate gene causing the disease. PTH1R Arg150Cys variant may contribute to the disease phenotype. SGK3 promoter deletion should be considered in patients with late-onset genetic hypophosphatemic rickets.

**Supplementary Information:**

The online version contains supplementary material available at https://doi.org/10.1186/s13023-026-04335-0.

## Introduction

Hereditary hypophosphataemic rickets (HR) is a group of renal phosphate wasting disorders with an incidence of 3.9-5 per 100,000 live birth per year [1, 2]. The X-linked HR (OMIM #307800), caused by an inactivating mutation in the *PHEX* gene (phosphate regulating gene with homologies to endopeptidases, is the most common hereditary form of HR representing about 80% of all the cases [3]. The remaining cases of HR are caused by different underlying genetic defects: *FGF23*, *FAM20C*, *DMP1*,* ENPP1*,* CLCN5*, *SGK3*, *SLC9A3R1*,* SLC34A1* or *SLC34A3*. For example, a gain-of-function mutation in the *FGF23* gene (fibroblast growth factor 23) leads to autosomal dominant HR (OMIM #193100) [4]; an inactivating mutation in the *DMP1* gene (dentin matrix acidic phosphoprotein 1) or in the *ENPP1* gene (ectonucleotide pyrophosphatase/phosphodiesterase 1) causes autosomal recessive HR-1(ARHR1, OMIM# 241520) or autosomal recessive HR-2 (ARHR2, OMIM#613312), respectively [5, 6]; an inactivating mutation in the CLCN5 gene (chloride channel 5) results in X-linked recessive HR (XLRHR or Dent disease 1, OMIM# 300009) [7, 8]; an inactivating mutation in the *SLC34A3* gene (sodium-dependent phosphate cotransporter 2 C) causes hereditary HR with hypercalciuria (OMIM# 241530) [9]; an inactivating mutation in the *SLC34A1* gene (sodium-dependent phosphate cotransporter 2 A) or in the *SLC9A3R1* gene (sodium-hydrogen exchanger regulatory factor1) results in hypophosphatemic rickets with nephrolithiasis and osteoporosis type 1 (NPHLOP1, OMIM#612286) or hypophosphatemic rickets with nephrolithiasis and osteoporosis type 2 (NPHLOP2, OMIM#612287), respectively [10, 11].

Maintaining physiological phosphate balance is critical for bone health. PTH, 1,25(OH)2D, and FGF23 play important roles in maintaining phosphate homeostasis by regulating intestinal absorption and renal reabsorption. Both PTH and FGF23 lower serum phosphate by decreasing its renal reabsorption, whereas 1,25(OH)2D increase serum phosphate by increasing intestinal phosphate absorption and renal phosphate reabsorption. In turn, serum phosphate regulates the production of PTH, 1, 25(OH)_2_D, and FGF23 by feedback mechanisms [12, 13].

Although underlying genetic defects could be detected in most HR patients, there are still small number of HR patients whose genetic defects could not be found [14, 15], indicating possible existence of novel HR-causing genes. Indeed, we have recently identified a mutant *SGK3* as one of genetic defects causing autosomal dominant hypophosphatemic rickets [16]. In the present study, we investigated a large Turkish family where a boy developed late-onset hypophosphatemic rickets. We found a novel promoter deletion of *SGK3* and a previously reported mutant *PTH1R* (parathyroid hormone 1 receptor) in the patient.

## Patient

**Fig. 1:**
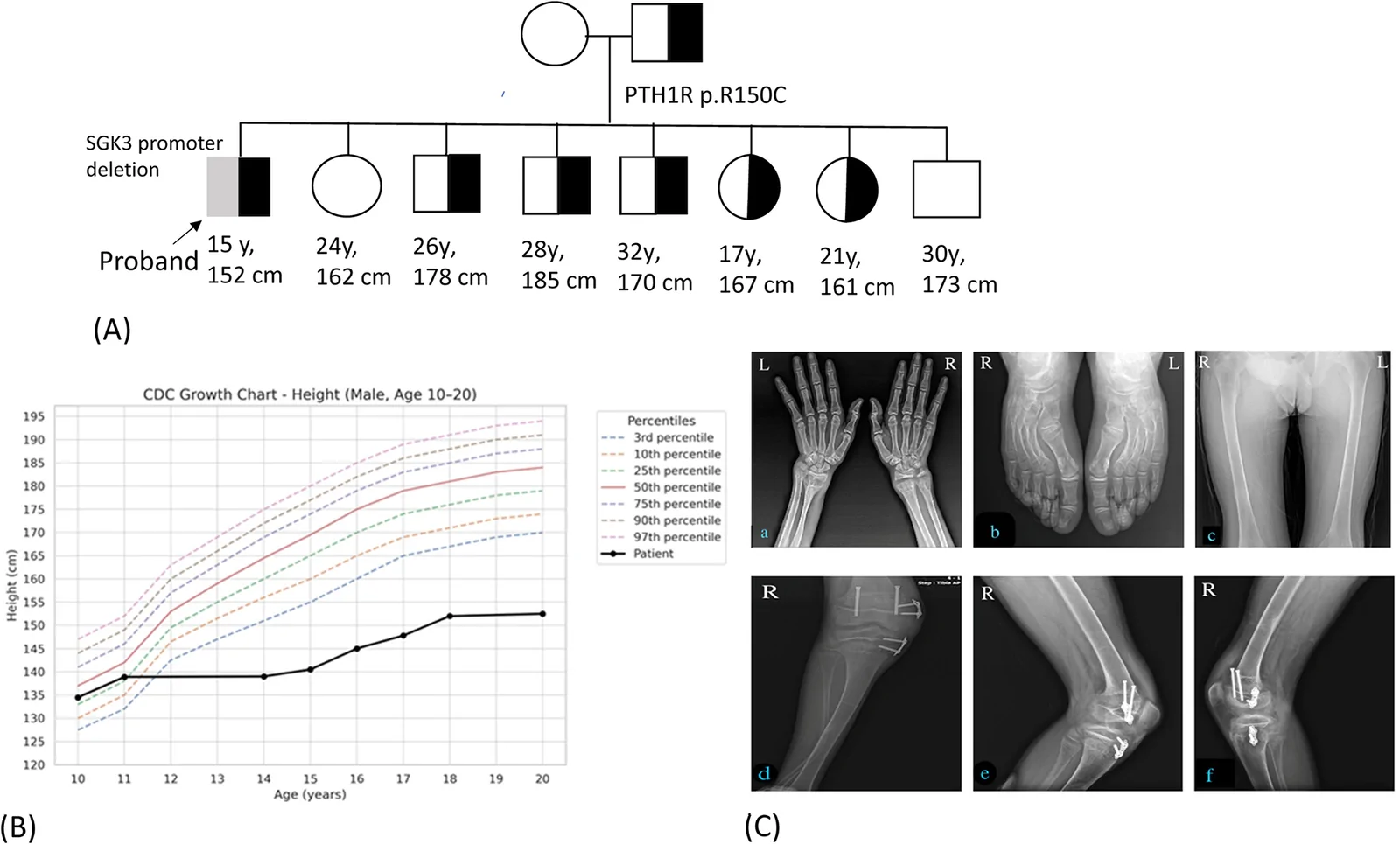
Patient with late-onset hypophosphataemic rickets. (**A**) Pedigree of a large family with one affected boy. The proband presented to the pediatric endocrinology outpatient clinic at 14 years and 7 months old and carries an inherited *PTH1R* p.Arg150Cys variant and a de novo *SGK3* promoter deletion. (**B**) Growth chart of the patient. His growth curve was normal until 11 years old. (**C**) Radiographic findings in the proband with hypophosphataemic rickets. (**a-c**) Hand-wrist, foot, and lower limb radiographs revealed bilateral and symmetrical rachitic changes, including metaphyseal widening, irregularity, and cortical/subcortical resorptive changes. (**d-f**) Postoperative radiographs of the right knee showing partial epiphyseal redirection surgery performed at 17 years of age to correct the genu valgum deformity

The proband and nine of his family members (7 siblings) were recruited for this study (Fig. 1A). The proband was of Kurdish ethnicity and was born to non-consanguineous parents. The study was approved by the Institutional Review Board, and written informed consent was obtained from parents or guardians. The proband is a 15-year-old boy who exhibited normal development until age 11 (Fig. 1B), when he developed widening of both wrists and bowing of both legs. He was referred to the pediatric endocrinology clinic for short stature and progressive leg deformity. Patient demographics, clinical features, laboratory results, skeletal findings, and treatment interventions were summarized in Table 1.

**Table 1:** Clinical, biochemical, and treatment interventions in the proband

Category	Parameter	Patient	Reference / Notes	Intervention / Response
Demographics	Age / Sex	15 / M	–	–
	Ethnicity / Origin	Turkish	–	–
Anthropometrics	Height (cm, SDS)	137.3 (-2.9)	–	19 yrs: 151.6 (-3.5 SD)
	Weight (kg, SDS)	26.3 (-4.6)	–	19 yrs: 38.2 (-5.1 SD)
Skeletal Findings	Physical exam	Metaphyseal widening at wrists, genu valgum, elbow contractures	–	Partial knee epiphyseal redirection surgery at 17 yrs
	Radiographs	Widening of metaphyses, cortical resorption, bowing of legs	–	–
	Bone mineral density (Z-score)	Lumbar spine L1–L4: -0.8	Normal ≥ -1; Osteopenia − 1 to -2.5; Osteoporosis ≤ -2.5	–
Biochemical Profile	Serum phosphorus (mg/dL)	1.0	2.5–4.5	Burosumab 1 mo: 1.5
	Serum calcium (mg/dL)	9.3	8.8–10.6	–
	Alkaline phosphatase (U/L)	1226	108–317	Burosumab 1 mo: 926
	PTH (pg/mL)	43	10–65	–
	25-hydroxyvitamin D (ng/mL)	23.1	20–100	–
	1,25-dihydroxyvitamin D (pg/mL)	22.2	17.4–93.5	–
	cFGF23 (RU/mL)	242	≤ 230	–
	Urine Ca/Cr (mg/mg)	0.09	< 0.2	–
	TRP (%)	84	> 85	–
	TmP/GFR (mg/dL)	1.1 and 1.7	2.6–3.9	Burosumab 1 mo: 1.5
Growth & Interventions	GH therapy	–	–	0.35 mg/kg/week for 6 mo; height velocity 9.1 cm/yr (+ 2.4 SD), height SD -4.2 → -3.4; discontinued due to funding
	Phosphorus + calcitriol	–	–	Initial therapy, limited adherence
	Burosumab	–	–	Started 1 mg/kg SC q2w, increased to 1.25 mg/kg q2w; after 1 mo: phosphate ↑ 0.96→1.5, ALP ↓ 1442→926, TmP/GFR ↑ 0.96→1.5; improved bone pain

Notes: SDS: standard deviation score, TRP: tubular reabsorption of phosphate, TmP/GFR: tubular maximum reabsorption of phosphate / glomerular filtration rate, Urine Ca/Cr: urine calcium (mg/dL) to creatinine ratio, ALP: alkaline phosphatase, Burosumab response reported after 1 month of therapy

On examination, he had swelling of the ankles and wrists, elbow contractures, and severe genu valgum. His weight was 26.3 kg (-4.6 SD) and height 137.3 cm (-2.9 SD), with a sexual maturity rating of 3. The parents were unrelated, with heights of 179 cm (father) and 162 cm (mother). The parents and his siblings were healthy, with no history of short stature, lower-limb deformity, bone pain, fatigue, or other features suggestive of hypophosphataemic rickets. The proband was the only member with short stature in the family. Radiographs revealed metaphyseal widening and bowing of the legs, consistent with rickets (Fig. 1C). Initial therapy with phosphorus and calcitriol showed limited adherence, partly due to pandemic-related disruptions. At 17 years, he underwent partial knee epiphyseal redirection surgery and a 6-month course of growth hormone, which temporarily improved height velocity. At 19 years, burosumab therapy was initiated, resulting in early biochemical improvement and subjective reduction in bone pain.

### Methods

**Genomic DNA isolation.** Genomic DNA from peripheral blood leukocytes was isolated using the Gentra Blood Kit (Qiagen Corp, CA).

#### DNA amplification and sequencing

DNA samples were first analyzed for mutations in all the coding exons and intron-exon boundaries of *PHEX* and *FGF23* genes. PCR primers and conditions were described previously [17, 18]. The resulting PCR products were directly sequenced using an automated ABI PRISM 3700 sequencer (Foster City, CA).

#### Next-generation exome sequencing

Genomic DNA from peripheral blood leukocytes was extracted and libraries were constructed for exome enrichment and capture using an Agilent’s SureSelect V5 Whole Exome Capture kit according to the manufacturer’s instructions (Santa Clara, CA, USA). NGS was performed on Illumina HiSeq 2000 with 50x coverage by standard Illumina protocol (San Diego, CA, USA) [19]. The *PTH1R* variant was verified by PCR-Sanger sequencing using two primer pairs: *PTH1R*-F: 5’-CAAGGAGGCTGAGACTTGGA-3 and *PTH1R-R*: 5’-CTGGAGGAGTCAAGGTCAGG-3’ with following PCR conditions: denaturation 95 C° for 1 min, annealing 58 C° for 1 min, and extension 72 C° for 1 min (35 cycles).

#### Next-generation whole genome sequencing (WGS)

Whole-genome sequencing libraries were constructed and then sequenced using DNBSEQ™ sequencing technology platforms (BGI Genomics’ Human Whole Genome Sequencing services) with 30X coverage and 90 Gb data. Copy number variation in the genomic DNA was analyzed by CNVnator. The *SGK3* promoter deletion was verified by PCR using two primers flanking the breakpoint: SGK3-F: 5’-TGTAGAGACGGTGTATCATC-3’ and *SGK3*-R: 5’-GCCCAAGTTGGTCTTGAACT-3’ with the following PCR conditions: denaturation 95 C° for 1 min, annealing 60 C° for 1 min, and extension 72 C° for 1 min (35 cycles). The PCR products were verified by Sanger sequencing. The verification of heterozygosity in the promoter deletion was performed using two primers within the deleted region: *SGK3*-F1: 5’-TGCAGAGGAATTACATAGGAT-3’ and *SGK3*-R1: 5’-CCGCCACCGCGCCCGGCTAAT-3’ with the same PCR conditions mentioned above.

#### The filtration strategy of exome and whole genome sequencing

Variants with minor allele frequency > 1% were excluded, and rare coding, splice-site, or promoter variants predicted to affect protein function were prioritized. Candidate variants were evaluated for segregation with affected individuals, functional relevance, and known disease associations. Putative pathogenic variants were validated by Sanger sequencing.

**In-silico analysis of the transcription factor binding sites in the SGK3 promoter region.** The analysis was performed using ConTra v3, which predicts conserved transcription factor binding sites across multiple species [20].

## Results

### PCR amplification and sanger sequencing for *PHEX* and *FGF23* mutations

Given elevated serum FGF23 in the patient, we first screened for *PHEX* and *FGF23* mutations by PCR-sequence analysis. No mutation was detected in both genes.

### Exome sequencing for genetic defects

**Fig. 2:**
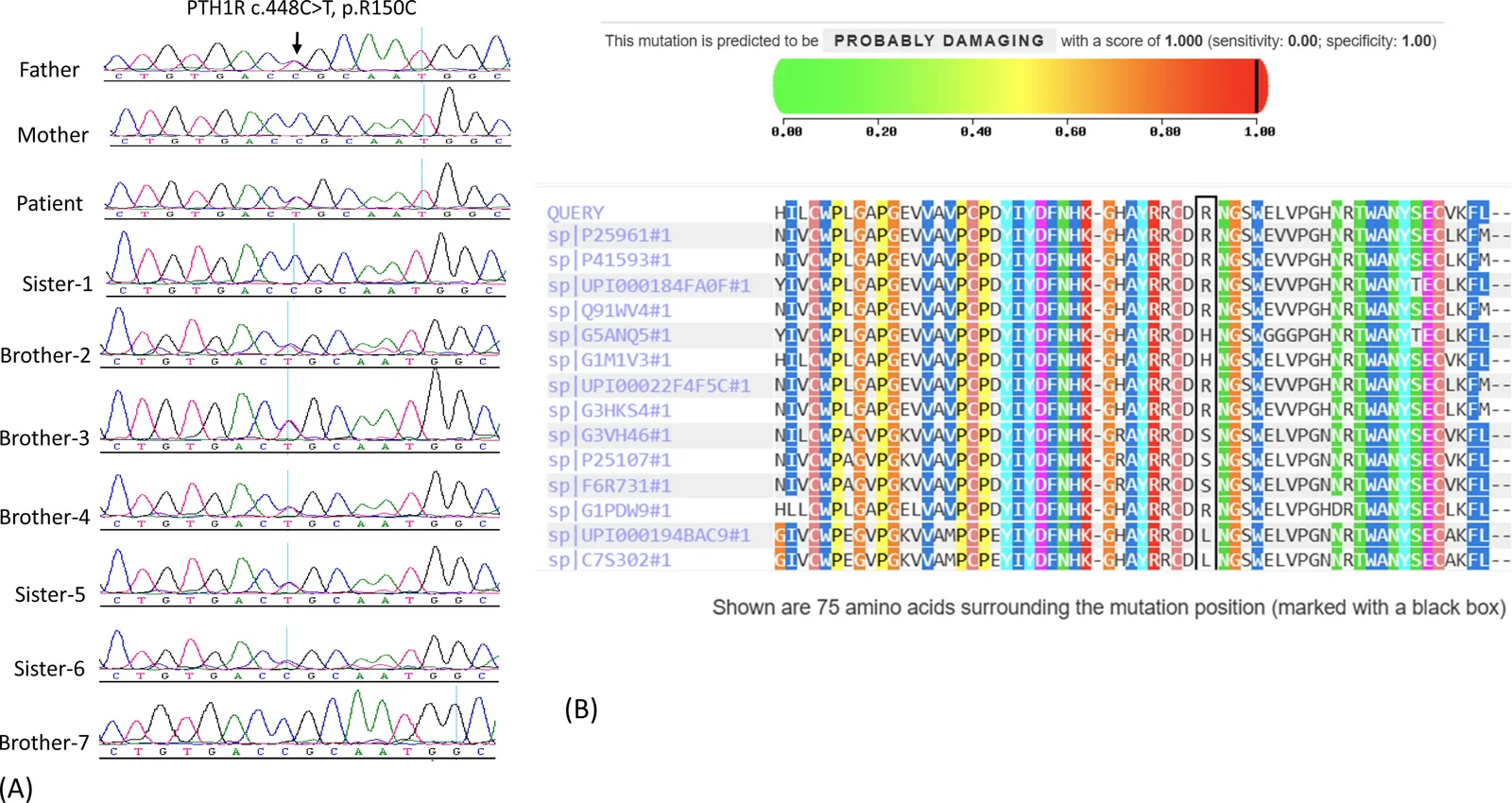
Sequence analysis of *PTH1R* c.448 C > T in the patient and his family members. (**A**) Representative electropherograms are shown. A heterozygous c.448 C > T was present in the patient, unaffected father and 5 normal siblings. The variant is indicated by an arrow. (**B**) PolyPhen-2 prediction of p.Arg150Cys variant and sequence alignment among different species

The negative *PHEX* and *FGF23* mutation results suggest possible existence of a novel genetic defect. We performed exome-sequencing of the patient and his entire family members. We could not found any mutations in the genes known to cause HR such as *FAM20C*, *DMP1*,* ENPP1*,* CLCN5*, *SGK3*, *SLC9A3R1*,* SLC34A1* or *SLC34A3*. Interestingly, a heterozygous variant c.448 C > T (p.Arg150Cys) in the *PTH1R* gene was found in the patient, his unaffected father and 5 normal siblings (Fig. 2). All other family members carrying the *PTH1R* p.Arg150Cys variant were clinically unaffected, showing no features of rickets or phosphate-wasting disorders. The variant was previously reported in human enchondromatosis and caused enchondroma-like lesions in transgenic mice [21]. Given the variant in the *PTH1R* gene was also present in the normal family members, it is unlikely the genetic cause of the disease. The patient may carry another pathogenic variant that is exclusively present in the patient and not in the normal family members.

### WGS for genetic defects

**Fig. 3:**
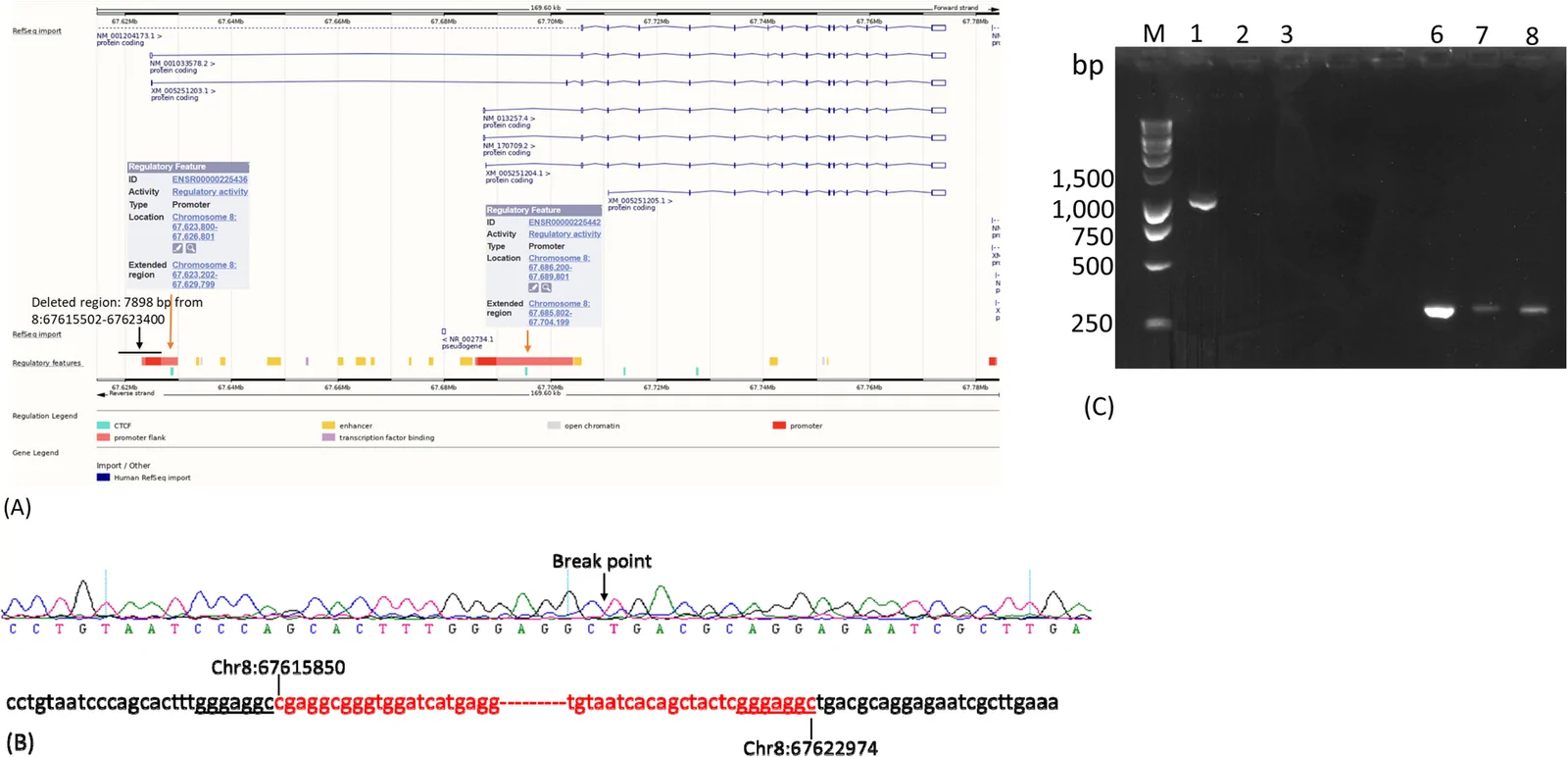
A large deletion in the *SGK3* distal promoter. (**A**)*SGK3* has two promoters indicated by a red arrow. The proximal promoter drives basal expression of SGK3. The distal promoter contains ERE and ARE and is estrogen- or androgen-inducible. (**B**) The breakpoint point of SGK3 promoter deletion. The deletion breakpoint is indicated by an arrow. The nucleotide sequence surrounding the deletion is shown. The deleted region is highlighted in red. The potential nucleotide sequence for homologues recombination is underlined. The 7124 bp deletion is located in the distal promoter region and is 1679 bp from first exon of *SGK3* (NM_001033578.3). (**C**) Agarose gel electrophoresis of PCR products flanking the breakpoint. The expected 976 bp truncated fragment was amplified only in the patient’s DNA, and not found in his parents, thus a de novo deletion (lane 1–3). A 360 bp fragment was amplified in both patient and his parents using primers in the deleted region (lane 6–8), indicating a heterozygous deletion in the patient. M: 1 kb DNA ladder from Promega, Lane 1 and 6: patient, Lane 2 and 7: father, Lane 3 and 8: mother

Since gross deletions and insertions/duplications was frequently reported in the *PHEX* gene (46/454, 10%, HGMD) and occasionally in the *SLC34A1* (2/30, 6.6%), we next performed genome-wide copy number analysis of the patient by WGS to search for deletions or amplifications in the disease-causing genes that might be missed by exome sequencing. Although no deletion or amplification was detected in the *PHEX* or *SLC34A1* gene, a 7898 basepair (bp) deletion was identified in the *SGK3* distal promoter region (chromosome 8:67615502–67623400, GRCh37.p13, Fig. 3A). The normalized read depth was 0.7132 by CNVnator, which was considered as a heterozygous deletion (< 0.25 imply homozygous deletion while 0.25–0.75 imply heterozygous deletion). The deletion was predicted to be located 1253 bp from the first exon of *SGK3* (NM_001033578.3). We next tried to determine the breakpoint of the deleted region by PCR amplification of mutant allele using two flanking primers. The wild-type allele could not be amplified by PCR due to large size of DNA fragment (more than 7 kb). Sanger sequencing of the mutant DNA fragment revealed that the deletion was 774 bp smaller than expected 7898 bp and was located 1679 bp from the first exon of *SGK3* (Fig. 3B), indicating WGS results still need to be verified by Sanger sequencing. Interestingly, two sequence repeats (gggaggc) were found in the breakpoint region (Fig. 3B), which may be involved in the homologous recombination leading to the deletion of distal promoter region. Furthermore, we tested whether the deletion was present in the parents of the patient. As shown in Fig. 3C, the expected 976 bp mutant allele was amplified only in the patient’s DNA, and not found in his parents, thus a de novo deletion. A 360 bp fragment was amplified in both patient and his parents using primers in the deleted region, indicating a wild-type allele is present in the patient.

## Discussion

The present study describes a large family with late-onset hypophosphatemic rickets (HR), which did not have disease-causing mutations in known HR-associated genes after exome sequencing. Notably, a large deletion in the *SGK3* promoter region was identified through whole genome sequencing, which could be the genetic basis for the disease in this family. Additionally, the patient carried a previously reported variant, c.448 C > T (p.Arg150Cys), in the *PTH1R* gene. This specific variant has been linked to enchondromatosis (Ollier disease, Maffucci syndrome) in two out of six reported cases and has been shown to cause enchondroma-like lesions in transgenic mice [21]. The combination of the *SGK3* promoter deletion and the *PTH1R* Arg150Cys variant may contribute to the development of late-onset HR in this family. The study could potentially offer new insights into the genetic and molecular mechanisms of late-onset HR and related skeletal disorders.

*SGK3* is a novel regulator of renal phosphate transport [22]. In *sgk3* knockout mice, urinary phosphate excretion is increased with reduced bone density and decreased plasma levels of FGF23, and 1.25 (OH) 2D3 [22]. We previously reported a novel c.979 − 96 T > A mutation in the *SGK3* gene in a large kindred with 5 HR patients [16]. The present study identified another novel mutation in the distal promoter region of *SGK3* in a case with late-onset HR. The identification of a late-onset form of HR with a mutation in the *SGK3* promoter region is particularly interesting given that late-onset HR has not been reported in the literature. Late-onset HR could suggest a form of the disease that manifests later in life, which may be due to gradual disruption of *SGK3* function.

*SGK3* has two main promoter regions: the proximal and distal promoter regions [23, 24]. The proximal promoter region drives basal expression of *SGK3* and lacks estrogen response element (ERE) or androgen response element (ARE). The distal promoter region contains both ERE and ARE and is inducible by estrogen or androgen, meaning its activity is enhanced in response to hormonal changes, such as during puberty. The consensus ARE sequence is comprised of two 6-bp half-sites separated by a 3-bp spacer: 5’-A/GGA/TACA (5’ half site) NNN (where N is any nucleotide) TGTTCT (3’ half site)-3’ [25, 26] whereas the consensus ERE sequence is a 13 bp palindromic inverted repeat: 5′-GGTCAnnnTGACC-3′. The deleted distal promoter region contains 3 putative AREs (consensus nucleotide is in italic): 5’-*AGT*T*CA*cacCA*TTCT*-3’, 5’-*GGT*T*CA*cgcCA*TTCT*-3’, and 5’-*GGT*T*CA*cgcCA*TTCT*-3’. They are 75% identical to the canonical ARE sequence (83% similarity to 5’ half site and 67% to 3’ half site of consensus sequence). The experimentally validated *SGK3* ARE (5’-*AGAAC*Ttcc CC*TTCT*-3’) is also 75% identical to the canonical ARE [24]. The 3’ half site of these putative AREs is 83% identical to the experimentally validated *SGK3* ARE (only 1 bp mismatch), indicating they may be involved in androgen-mediated induction of *SGK3* expression. The deleted region also contains 1 putative ERE (consensus nucleotides are in italic): 5’-*GGTCA* ggc*TG*GT*C*-3’, which shows 80% similarity to the canonical ERE or experimentally validated *SGK3* ERE (5’-*GGTCA*tcc*TGA*A*C*-3’), further linking the deleted region to hormonal regulation. The loss of both AREs and ERE in the deleted distal promoter region likely disrupts the hormonal regulation of *SGK3*. This disruption may have contributed to the patient’s late-onset HR, coinciding with the onset of puberty at age 11. The activity of the proximal basal promoter may have been insufficient to compensate for the loss of the distal promoter, particularly given the increased developmental demand for SGK3-mediated renal phosphate transport during pubertal growth. Furthermore, we performed additional in silico analyses of the deleted distal promoter region. Additional putative estrogen receptor (ER) and androgen receptor (AR) binding sites were identified (supplemental Table 1) as well as binding sites for other transcription factors such as FOXP1, ZFH-2, UBX, SOC1, LMX1A, YOX1, Lhx4, ARX, FOXP1, IRF1, PROP-1, FOXC1, FOXA2, FOXB1, and XFD-3 (supplemental Table 2), While these analyses do not provide direct functional validation, they support the hypothesis that the deleted distal promoter region contains regulatory elements that may contribute to the transcriptional regulation of SGK3. The direct association between the SGK3 promoter deletion and the observed phenotype remains speculative until detailed functional analyses are performed.

PTH1R is essential to induce FGF23 production and maintain systemic mineral ion homeostasis [27]. Constitutively active *PTH1R* mutations result in ligand-independent cAMP accumulation and Jansen’s metaphyseal chondrodysplasia characterized by abnormal endochondral bone formation, markedly elevated serum levels of FGF23, hypercalcemia and hypophosphatemia despite normal/low levels of PTH and 1,25(OH)2 vitamin D [28]. Unlike *PTH1R* activating mutants His223Arg or Thr410Pro, which causes Jansen’s metaphyseal chondrodysplasia and induce four to six times higher basal cAMP than wild-type receptors [29, 30], *PTH1R* Arg150Cys is weaker in receptor activation and causes only 2-fold increase in basal cAMP level [21]. This may explain the heterozygous variant is not sufficient to cause hypophosphatemia or bone deformity, but may contribute to the mild elevation of serum FGF23 and trigger the late-onset manifestation at the start of puberty. It is therefore tempting to propose a “two-hit” or threshold-based model. While the p.Arg150Cys variant in *PTH1R* is unlikely to be the primary driver of disease, it may function as a genetic modifier that lowers the pathogenic threshold, thereby contributing to disease manifestation in the context of *SGK3* deletion. This combinatorial effect may have become clinically apparent during puberty, a period characterized by increased hormonal and skeletal remodeling demands.

The patient had severe growth deficit. It is possible that chronic malnutrition or malabsorption could have compounded the genetic defect to exacerbate the clinical phenotype. However, the patient exhibited normal growth and nutritional status until approximately 11 years of age (Fig. 1B and C). Given this normal growth trajectory prior to disease onset, chronic malnutrition or generalized malabsorption is unlikely to be the primary cause of the severe growth deficit. Instead, the marked growth impairment observed after 11 years of age is more consistent with disease-related phosphate wasting and impaired phosphate absorption associated with the underlying genetic defect, which would adversely affect bone mineralization and growth.

### Limitations of the study

This study has several limitations. First, the findings are based on a single family, which may limit the generalizability of the observations. Second, although a deletion affecting the *SGK3* promoter was identified and is a plausible contributor to the phenotype, functional studies confirming its effect on SGK3 expression or downstream signaling were not performed. Future studies, including functional validation and identification of additional cases, will be necessary to further clarify the pathogenic role of this variant.

In summary, we identified a novel deletion in the distal promoter region of *SGK3* in a patient with late-onset autosomal dominant HR. This promoter deletion may disrupt regulatory elements controlling *SGK3* transcription and could contribute to the delayed onset of disease manifestation. Our findings expand the current understanding of HR, which is typically considered a disorder presenting earlier in life. In addition, this study highlights the value of WGS in identifying non-coding regulatory variants and structural alterations that may not be detected by exome sequencing, thereby facilitating the resolution of complex or previously unsolved genetic cases.

## Supplementary Information

Below is the link to the electronic supplementary material.


Supplementary Material 1



Supplementary Material 2


## Data Availability

All the data are included in the manuscript. Materials are available upon request.
