# Supplementary material for: SGK3 promoter deletion in late-onset hypophosphatemic rickets, a possible genetic cause of the disease
Source: Orphanet J Rare Dis. 2026 Mar 30;21:310. doi: 10.1186/s13023-026-04335-0 (PMC13528025; doi:10.1186/s13023-026-04335-0)
Supplement: Supplementary file 1 [file 13023_2026_4335_MOESM1_ESM.docx]

**Supplemental Table 1. Visualization of predicted AR and ER binding sites** **and alignment among different species.**

Genomic position: 8:67615502-67623400; Stringency: core = 0.95, similarity matrix = 0.85,

Position: chr8:67615801-67615933, predicted estrogen (green) and androgen (pink and orange) receptor binding sites.

Position: chr8:67616110-67616332, predicted estrogen (green) and androgen (pink and orange) receptor binding sites.

Position: chr8:67617535-67617697

Position: chr8:67617697-67617815

Position: chr8:67618709-67619049

Position: chr8:67619049-67619375

Position: chr8:67619572-67620194

Position: chr8:67620404-67620464

Position: chr8:67621654-67621803

Position: chr8:67622434-67622551

Position: chr8:67622641-67622728

Position: chr8:67622838-67623053
