# Supplementary material for: SGK3 promoter deletion in late-onset hypophosphatemic rickets, a possible genetic cause of the disease
Source: Orphanet J Rare Dis. 2026 Mar 30;21:310. doi: 10.1186/s13023-026-04335-0 (PMC13528025; doi:10.1186/s13023-026-04335-0)
Supplement: Supplementary file 2 [file 13023_2026_4335_MOESM2_ESM.docx]

**Supplemental Table 2. Visualization of predicted transcription factor binding sites and alignment among different species.**

Genomic position: 8:67615502-67623400; Stringency: core = 0.95, similarity matrix = 0.85

Different predicted transcription factor binding sites are highlighted in different colors.

FOXP1, ZFH-2, UBX, SOC1, LMX1A, YOX1,  Lhx4, ARX, FOXP1, IRF1,   PROP-1, FOXC1,   FOXA2,   FOXB1,   XFD-3

Position: chr8:67615545-67615577

Position: chr8:67615577-67615750

Position: chr8:67615750-67615801

Position: chr8:67616110-67616332

Position: chr8:67616577-67616643

Position: chr8:67616643-67616692

Position: chr8:67616692-67616813

Position: chr8:67616853-67616906

Position: chr8:67616906-67616934

Position: chr8:67617116-67617139

Position: chr8:67617207-67617241

Position: chr8:67617311-67617392

Position: chr8:67617444-67617471

Position: chr8:67619472-67619547

Position: chr8:67619547-67619572

Position: chr8:67620328-67620357

Position: chr8:67620404-67620464

Position: chr8:67620495-67620551

Position: chr8:67621518-67621559

Position: chr8:67621803-67621901

Position: chr8:67622135-67622246

Position: chr8:67623053-67623094
